# LoFTK: a framework for fully automated calculation of predicted Loss-of-Function variants and genes

**DOI:** 10.1186/s13040-023-00321-5

**Published:** 2023-02-02

**Authors:** Abdulrahman Alasiri, Konrad J. Karczewski, Brian Cole, Bao-Li Loza, Jason H. Moore, Sander W. van der Laan, Folkert W. Asselbergs, Brendan J. Keating, Jessica van Setten

**Affiliations:** 1 Department of Cardiology, Division Heart and Lungs, University Medical Center Utrecht, University of Utrecht, Heidelberglaan 100, 3584 CX Utrecht, Netherlands; 2 Medical Genomics Research Department, King Abdullah International Medical Research Center, King Saud Bin Abdulaziz University for Health Sciences, Ministry of National Guard Health Affairs, Riyadh, Saudi Arabia; 3 Program in Medical and Population Genetics, Broad Institute of MIT and Harvard, Cambridge, MA USA; 4 Analytic and Translational Genetics Unit, Massachusetts General Hospital, Boston, MA USA; 5 Bioinformatics Core, Harvard Medical School, Boston, MA USA; 6 Perelman School of Medicine, University of Pennsylvania, Philadelphia, PA USA; 7 Department of Computational Biomedicine, Cedars-Sinai Medical Center, Los Angeles, CA USA; 8 Central Diagnostic Laboratory, Division Laboratories, Pharmacy, and Biomedical Genetics, University Medical Center Utrecht, University of Utrecht, Utrecht, Netherlands; 9 Department of Cardiology, Amsterdam University Medical Centers, University of Amsterdam, Amsterdam, Netherlands; 10 Health Data Research UK and Institute of Health Informatics, University College London, London, UK

**Keywords:** Loss-of-Function variants, Knockout genes, Compound heterozygotes, Human genetic

## Abstract

**Background:**

Loss-of-Function (LoF) variants in human genes are important due to their impact on clinical phenotypes and frequent occurrence in the genomes of healthy individuals. The association of LoF variants with complex diseases and traits may lead to the discovery and validation of novel therapeutic targets. Current approaches predict high-confidence LoF variants without identifying the specific genes or the number of copies they affect. Moreover, there is a lack of methods for detecting knockout genes caused by compound heterozygous (CH) LoF variants.

**Results:**

We have developed the Loss-of-Function ToolKit (LoFTK), which allows efficient and automated prediction of LoF variants from genotyped, imputed and sequenced genomes. LoFTK enables the identification of genes that are inactive in one or two copies and provides summary statistics for downstream analyses. LoFTK can identify CH LoF variants, which result in LoF genes with two copies lost. Using data from parents and offspring we show that 96% of CH LoF genes predicted by LoFTK in the offspring have the respective alleles donated by each parent.

**Conclusions:**

LoFTK is a command-line based tool that provides a reliable computational workflow for predicting LoF variants from genotyped and sequenced genomes, identifying genes that are inactive in 1 or 2 copies. LoFTK is an open software and is freely available to non-commercial users at https://github.com/CirculatoryHealth/LoFTK.

**Supplementary Information:**

The online version contains supplementary material available at 10.1186/s13040-023-00321-5.

## Introduction

Loss-of-function (LoF) variants are determined to have a critical effect on gene function by inactivating protein-coding genes [1]. Remarkably, recent analyses of the human genome have uncovered that individuals harbor many dozens of LoF variants, including stop-gained, frameshift variants and splice site disruptions [2, 3]. On average, LoF variants are deleterious, and thus usually tend to be found at very low frequencies in the human population. These variants can have a profound impact on the gene transcripts and translated proteins. The association of LoF variants with complex diseases and phenotypic traits may lead to the discovery and validation of novel therapeutic targets [3]. However, hundreds of LoF variants are functionally neutral with no detectable influence on phenotypes [4, 5].

Several difficulties emerge when evaluating LoFs on a broad scale. False positives in the prediction of LoF variants can arise due to artifacts that may occur during genotype calling, mapping, imputation and annotation [3]. To annotate high-confidence (HC) LoF variants only, Loss-Of-Function Transcript Effect Estimator (LOFTEE) [6] can be used. LOFTEE is a plugin implemented in the Ensembl Variant Effect Predictor (VEP) [7] that imposes stringent filtering criteria to annotate HC LoF variants, eliminates nonsense mutations that are unlikely to impact protein function, and excludes LoF variants that are enriched with annotation artifacts.

However, LoF variants discovery can also be used to predict single-copy losses (heterozygous LoF variants) that inactivate a single copy of a gene, or two-copy losses that completely knockout a gene. Two-copy losses can be caused by homozygous and compound heterozygous (CH) LoF variants. CH variants appear when parents both donate a LoF-causing allele that locates at different loci in the same gene [8]. There is mounting evidence that CH LoF variants have a role in complex diseases. For example, both homozygous and CH LoF variants have been found to increase the risk of autism spectrum disorder [9, 10].

Current tools, such as LOFTEE and ALoFT, only annotate LoF variants and provide variant-level output [6, 11]. They do not identify genes and distinguish between single-copy and two-copy loss genes. Furthermore, the collection of available tools to identify and annotate LoF variants require in-depth computational skills impeding the usage by scientists less skilled in bioinformatics. As far as we are aware, no user-friendly, automated bioinformatics pipeline exists to identify CH LoF variants, and single-copy and two-copy LoF genes, and that also provides the necessary input for downstream (association) analyses. The development of a bioinformatics pipeline that automatically parses the VEP-LOFTEE result files in a single workflow to the input required for downstream analyses, would democratize the use of LoF data to a broader range of biomedical scientists that would only require limited bioinformatic skills.

**Fig. 1:**
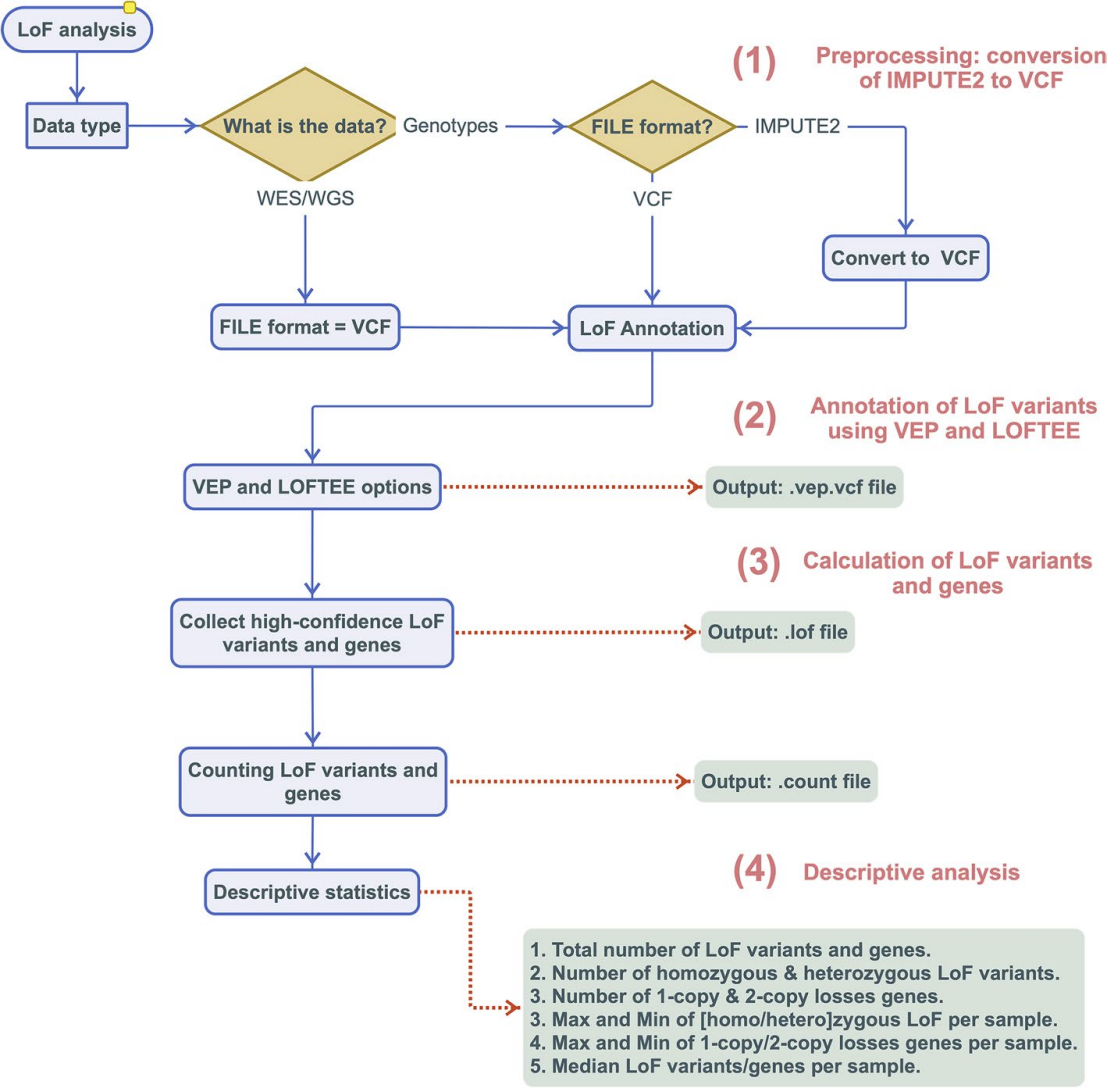
The workflow of LoFTK pipeline. Four steps involved in LoFTK; (1) preprocessing from IMPUTE2 to VCF, (2) LoF annotation and vep.vcf.file creation, (3) filtering HC LoF variants and counting LoF variants and genes, and (4) descriptive analysis

Here we present an open source tool, the Loss-of-Function ToolKit (LoFTK), which allows efficient and automated prediction of LoF variants and identifies genes that are inactive in one or two copies using genetic data derived through array-based genotyping imputed or whole-genome sequencing. LoFTK analyzes and parses genetic data in four steps as explained in the *Implementation* and depicted in Fig. 1; 1) Annotation of HC LoF variants from large-scale sequencing and array-based data using VEP and LOFTEE; 2) Identification of one-copy loss and two-copy loss of genes by parsing the CSQ field for each HC LoF variant in the VCF file as generated by VEP-LOFTEE; 3) Generation of a summary data for LoF-wide association analyses; and 4) Creating a statistical report describing the total number of LoF variants (homozygous, heterozygous and CH), LoF genes (single-copy and two-copy loss), and the average, minimum and maximum numbers of LoF variants and genes per sample. LoFTK aids to bridge the divide between computational scientists and wet-lab based trained biomedical scientists by simplifying the processing of VCF-based data to a useful format for downstream analyses in statistical tools like R.

## Implementation

### Main workflow

The LoFTK workflow consists of 4 analytical steps visualized in Fig. 1 and described below.

#### Preprocessing: conversion of IMPUTE2 to VCF

The first step depends on the input data formats. Two common file formats are permitted as inputs; IMPUTE2 [12, 13] output format and the Variant Call Format (VCF). The input data has to contain phased genotypes for distinguishing compound heterozygotes from two variants on the same allele. LoFTK uses two quality metrics for imputed genotypes: the imputation quality (info score) and imputed allele probability. The imputation quality contains values between 0 and 1, where higher values mean that a variant has been imputed with more certainty. Besides, imputation methods generate a probabilistic prediction of the missing genotypes, which stands for the likelihood of carrying genotypes combinations of A/A, A/B, and B/B in a particular individual. The supreme estimated genotype is the genotype that has the highest likelihood of being correct [14]. LoFTK has cut-off options to filter based on the optimal imputation quality metrics (Supplementary Material). After filtering, IMPUTE2 files are converted to VCF files. The VCF files that are generated from IMPUTE2 files or introduced directly by the user are applied as an input to the next step.

#### LoF annotation

The second step consists of annotation of LoF variants using VEP and LOFTEE. LOFTEE utilizes the Ensembl API framework to annotate HC LoF variants. LoFTK is highly customizable, with the ability to change VEP and LOFTEE flags in a configuration file. We designed LoFTK to be capable of processing data with *Homo sapiens* (human) genome assemblies GRCh37 and GRCh38, and it can easily be upgraded to future genome builds. The VEP will return results as VEP VCF format, which is similar to the input VCF, but in addition shows LoF information in the INFO field, such as LoF flags and LoF filtering outcome (high-confidence or low-confidence).

#### Calculation of LoF variants and genes

From the VEP output, the HC LoF variants are filtered, followed by parallel determination of homozygous and heterozygous LoF variants (Table 2) and allele frequencies, as well as the copy number loss (single-copy or two-copy) of LoF genes (Table 1). LoFTK recognizes CH LoF variants, which result in LoF genes with two copies losses. The LoF genes are extracted by parsing the CSQ field for each HC LoF variant in the VCF file that produced from the VEP. Optionally, LoFTK can be used to determine ‘mismatched genes’ between samples; these are genes that are active in one or two copies in one sample and completely inactive in the other sample. This feature helps study interactions between human genomes, for instance during pregnancy (maternal vs fetal genome) and after stem cell or solid organ transplantation (donor vs recipient genome).

**Table 1:** The output of predicted LoF variants from WES in UKBB. High-confidence LoF variants are listed in the first column, followed by their consequences in the second column. The third and fourth columns show frequencies of heterozygous and homozygous LoF variants, respectively. The rest of columns indicate the zygosity of LoF variants for each individual; 0 for not carrying LoF variant, 1 for heterozygous LoF variant and 2 for homozygous LoF variant

**SNP_ID**	**Consequence**	**heterozygous_LoF_frequency**	**homozygous_LoF_frequency**	Sample 1	Sample 2	Sample 3
chr19_52300416_CT_C	frameshift_variant	0.386	0.539	1	1	2
chr7_21543345_G_T	stop_gained	0.499	0.250	0	1	1
chr10_72508273_T_C	splice_acceptor_variant	0.425	0.450	2	1	0
chr1_3602477_AC_A	frameshift_variant	4.98E-06	0	1	0	0
chr7_21543345_G_T	stop_gained	0.499	0.250	0	2	1

#### Descriptive analysis

Finally, descriptive statistics of LoF variants are calculated, such as the total number of LoF variants, number of single-copy and two-copy LoF genes, and median of LoF variants per participant.

### Imputation quality threshold

The imputed genotype data provides two quality metrics: the INFO score and the imputed alleles probability. UK Biobank (UKBB) was used as the gold standard for determining the optimal quality metrics for obtaining the most genuine LoF variants from imputed genotypes data. We retrieved whole exome sequencing (WES) and array genotypes data from 4,476 randomly selected UKBB participants. Both data were phased using SHAPEIT2 [15] and array genotypes were imputed by IMPUTE2 [12, 13]. A combined reference panel from the 1000 Genome project phase 3 [4] and Genome of the Netherlands (GoNL) study [16] was used for phasing and imputation. We used LoFTK for LoF analysis in phased WES and three datasets of variants in imputed genotypes data. These subsets were divided based on variants with INFO scores above: 0.3, 0.6, 0.9. for each individual, predicted LoF variants in the WES were compared to LoF variants in each subset with considering the imputed allele probabilities ranging from 0.01 to 0.1 for that variant (Supplementary Fig. 1), in order to count the number of false negatives (average of LoF variants predicted in WES data but not in imputed data) and false positives (average of LoF variants predicted in imputed data but not in WES data).

### Validity of predicted CH LoF in trios

LoFTK is capable of annotating CH LoF variants, which introduce two inactive copies of a gene. To confirm the transmission of genuine CH LoF variants from parents to probands, we used trio-family genotype data from the Genome of the Netherlands (GoNL) cohort (Illumina Immunochip microarray SNP data) [16]. We performed a quality control step as preprocessing filtrations to impute genotypes data (Supplementary Material). We used the TOPMed imputation server [17] to impute untyped variants in 760 individuals from 250 families. LoFTK predicted LoF variants from imputed genotypes and we investigated transmission of CH LoF variants from parents to offspring.

### Exome sequences in UKBB

UKBB data were made available under the North West Multi-centre Research Ethics Committee (reference 11/NW/0382). UKBB data used in this study were obtained under application number 24711. We applied LoFTK on exome sequences of 200,643 UKB participants that were released in October 2020 [18]. We filtered participants exome data restricted to unrelated homogeneous white British population (Field IDs: 22,018 and 22,006) and removed singleton variants (MAC < 2). We phased exomes genotypes of 166,991 homogeneous white British participants using Eagle2 [19] followed by LoFTK analysis in order to identify CH knockout genes. Several genes were selected as positive controls that known to be associated with specific traits in UKBB [20]. We tested the association between these LoF genes and traits using linear regression for quantitative data and logistic regression for binary data with age, sex, and principal components 1–16.

## Results and discussion

### LoFTK software

LoFTK is a command-line tool that provides a robust computational workflow pipeline for predicting LoF variants from array-based (genotyped or imputed) and sequenced genomes, discovering genes that are inactive in 1 or 2 copies. LoFTK was developed using Perl and BASH scripting languages which make the code easily understandable, modifiable and extendable when needed. Instructions on how to install and run LoFTK as well as example datasets are publicly available at https://github.com/CirculatoryHealth/LoFTK. The code-setup of LoFTK is such that it is highly customizable through options and directories settings explained in the LoF.config file and GitHub README. It is designed to run as a command line program with user-friendly flags, which helps non-experts users to get quickly familiarized. LoFTK requires pre-installed tools, including BASH and Perl (> = version 5.10.1) which are commonly installed on Linux-based system, and the more specialized tools Ensembl VEP (https://github.com/Ensembl/ensembl-vep) and LOFTEE (https://github.com/konradjk/loftee) which both come with extensive installation documentation. We tested LoFTK on a computer cluster using CentOS 7 and managed by SLURM or SGE.

### Generation of LoF variants and genes

LoFTK uses the information present in large-scale sequencing and genotyping data to generate four files; two matrices of LoF variants and their respective genes, a list of LoF variants allele frequencies, and a report with descriptive statistics on the variants and genes. In the LoF variants matrix, the variants are represented as rows, and individuals are represented as columns. Each matrix's cell contains a number that represents the homozygous or heterozygous status of a given LoF variant for a given individual as shown in Table 1. Similarly, the columns in the LoF genes matrix define individuals except the rows represent the LoF genes, and each number in the matrix cell indicates that either the gene has no copy loss (0), single-copy loss (1) or two-copy loss (2) (Table 2). The frequencies in both matrices represent the frequency of heterozygous and homozygous LoF variants among the provided samples (Table 1), as well as the frequency of one-copy and two-copy LoF genes (Table 2). Finally, LoFTK generates information file with “.info” extension to show descriptive statistical report for predicted LoF variants and genes, such as the total LoF variants and genes, total heterozygous and homozygous LoF variants, total single-copy and two-copy LoF genes, and median of LoF variants and genes per participant.

**Table 2:** The output of predicted LoF genes from WES in UKBB. This table shows the predicted LoF gene ID and symbol in columns 1 and 2, respectively. The third column represents the frequency of single-copy loss gene, while the fourth represents the frequency of two-copy losses gene. The rest of columns indicate the number of copy losses for each individual; 0 for not carrying LoF gene, 1 for sigle-copy LoF gene and 2 for two-copy LoF genes

**gene_ID**	**gene_symbol**	**1_copy_LoF_frequency**	**2_copy_LoF_frequency**	Sample 1	Sample 2	Sample 3
ENSG00000198464	ZNF480	0.388	0.535	2	1	1
ENSG00000105877	DNAH11	0.502	0.251	1	0	1
ENSG00000152936	IFLTD1	0.013	0	0	1	0
ENSG00000039537	C6	4.47E-04	2.23E-04	0	0	0
ENSG00000221938	OR2A14	0	2.23E-04	0	0	0

### Imputation quality cut-off points

We assessed imputation quality metrics for obtaining the most genuine LoF variants in imputed genotype data by comparing existence of each predicted LoF variant between WES and three imputed datasets (INFO > 0.3, 0.6, 0.9) with considering the imputed allele probability cutoffs between 0.01 to 0.1 (see Sect. 2.2.).

LoFTK analysis for imputed dataset with INFO > 0.9 shows an optimal prediction of true LoF variants, because it has less false positive 2-copy LoF variants compared to the others (0.3 and 0.6) (Supplementary Fig. 1). However, choosing an optimal imputed allele probability was difficult due to the lack of apparent variations.

### CH LOF variants in trios

CH LoF variants occur when both parents donate a single LoF allele to proband at distinct loci within the same gene. We used trio-families from the GoNL [16] to evaluate the accuracy of obtaining two inactive copies in genes caused by CH LoF variants (see Sect. 2.3.).

We predicted LoF variants and genes in 250 families' imputed genotypes (760 individuals). We found 642 LoF variants affecting 571 genes (Table 3). In 164 probands, we identified 250 events of CH LoF variants producing 2-copy LoF genes. There were 240 (96%) true transmissions of CH LoF in parent-offspring, whereas there were 10 false transmissions.

**Table 3:** Predicted LoF variants and genes in the GoNL. LoF genes column shows numbers of total LoF genes, one copy inactive genes (1-copy) and two copies inactive genes (2-copy). LoF variants shows total number of predicted LoF variants, heterozygous and homozygous variants

	LoF genes	LoF variants
Total	571	642
1-copy	571	-
2-copy	196	-
Median 1-copy per individual	49	-
Median 2-copy per individual	21	-
Heterozygous	-	641
Homozygous	-	213
Median heterozygous per individual	-	54
Median homozygous per individual	-	21

### LoF variants and genes in ~ 200 K exomes from UKBB

We predicted LoF variants and genes in unphased exomes of 200,643 participants (mixed populations). We identified 398,377 heterozygous LoF variants affecting 17,796 genes and 2,383 homozygous LoF variants affecting 1,798 genes. Next, to determine CH variants, we phased exomes of 166,991 homogenous, unrelated white British individuals and found 16,464 1-copy LoF genes and 1,510 2-copy LoF genes. Of the 2-copy LoF genes, 481 were caused by homozygous variants only, 307 by CH variants only, and 722 by both homozygous and CH variants. To prove that we correctly identified homozygous and CH LoF genes, we examined nine LoF genes (as positive controls) that are known to be associated with specific traits. All of them showed a significant association and the expected direction of effect (Supplementary Table 2).

### Limitations

Some limitations of the current LoFTK version should be considered: LoFTK relies on preexisting methods for phasing, imputation, genotype calling, and variant effect prediction, which means results can be affected by errors generated by these software. Errors rate varies from one sequencing platform to another in the variant calling step, making it difficult to predetermine error rates. Lastly, predicting LoF variants and genes from unphased data will not allow the detection of CH LoF variants, which means users will have to input phased data to make full use of LoFTK.

## Conclusions

Prediction of LoF variants and genes provide important insight into the discovery of possible disease-causing mutations and potential therapeutic targets. LoFTK is easy to use and helps users to predict LoF variants from genotyped and sequenced genomes, identifying genes that are inactive in 1 or 2 copies, and providing summary statistics report describing the total number of LoF variants, LoF genes, and their average, minimum and maximum per sample. LoFTK is highly customizable and extra features for the identification of knockout genes in copy number variation (CNV) and predicting the pathogenicity of predicted LoF variants can be easily added.

## Supplementary Information

**Additional file 1: Supplementary Table 1.** False positive and false negative values from matching the LoF variants between exome and subgrouped imputed genotypes data*.***Supplementary Table 2.** Association of selected positive control LoF genes with UKBB phenotypes. **Supplementary Figure 1.** The workflow for achieving the optimal imputation quality measure.

## Data Availability

The data used in this article were provided by the UK Biobank under application (#24,711) and the Genome of the Netherlands under application number (#2,021,217). Access to these data can be achieved by request from UK Biobank (https://www.ukbiobank.ac.uk/enable-your-research/apply-for-access) and the Genome of the Netherlands (https://www.nlgenome.nl/menu/main/app-request).

## Abbreviations

LoF: Loss-of-Function
CH: Compound heterozygous
LoFTK: Loss-of-Function ToolKit
HC: High-confidence
LOFTEE: Loss-Of-Function Transcript Effect Estimator
VEP: Ensembl Variant Effect Predictor
VCF: Variant Call Format
GoNL: Genome of the Netherlands
UKBB: UK Biobank
WES: Whole exome sequencing
CNV: Copy number variation
