# Supplementary material for: LoFTK: a framework for fully automated calculation of predicted Loss-of-Function variants and genes
Source: BioData Min. 2023 Feb 2;16:3. doi: 10.1186/s13040-023-00321-5 (PMC9893534; doi:10.1186/s13040-023-00321-5)
Supplement: Supplementary file 1 — Additional file 1: Supplementary Table 1. False positive and false negative values from matching the LoF variants between exome and subgrouped imputed genotypes data.Supplementary Table 2. Association of selected positive control LoF genes with UKBB phenotypes. Supplementary Figure 1. The workflow for achieving the optimal imputation quality measure. [file 13040_2023_321_MOESM1_ESM.pdf]

Supplementary material

LoFTK: a framework for fully automated calculation of predicted Loss-of-Function variants and genes

Table of Contents

Sections **1**

Supplementary text **2**

Determining the optimal imputation quality threshold *2*

Compound heterozygous LoF variants *3*

Supplementary Tables **4**

Supplementary Figure **6**

References **7**

Supplementary text

Determining the optimal imputation quality threshold

LoFTK was developed to analyze any genetic data, such as (imputed) genotypes and (exome) sequencing data. The imputed genotype data provides two quality metrics, which are INFO score and imputed alleles probability. The quality metrics can be used for filtering imputation results per individual variant. We determined the optimal imputation quality metrics in order to extract only the most genuine LoF variants. We used whole exome sequencing (WES) data from UK biobank (UKBB) as a gold standard for evaluating the optimal quality metrics to obtain the most genuine LoF variants from imputed genotypes data. We extracted WES and genotypes data of 4,476 randomly selected UKBB participants. Genotypes were phased and imputed using SHAPEIT2 and IMPUTE2, respectively [1–3]. Phasing and imputation were performed using a combined reference panel from the 1000 Genome project phase 3 [4] and Genome of the Netherlands (GoNL) study [5]. The imputed genotypes were submitted to LoFTK using a range of info scores and imputed allele probabilities in order to obtain various numbers of LoF variants for each subset. On the other hand, the UKBB provided unphased WES data, thus we phased the data using SHAPEIT2 and then converted to VCF. We retrieved overlapped variants between phased exomes and imputed genotypes data (Supplementary Figure 1).

In the imputed genotype data, we analyzed LoF variants for three different datasets, where the first has INFO > 0.3, the second has INFO > 0.6 and the third has INFO > 0.9. We compared the existence of each LoF variant between each imputed genotypes dataset and WES with considering the imputed allele probabilities (0.01 – 0.1) for that variant (Supplementary Figure 1). For each individual, each LoF variant from the three imputed datasets was matched to WES, in order to count the false negative (average of LoF variants found in WES data but not in imputed data) and

false positive (average of LoF variants found in imputed data but not in WES data) (Supplementary Table 1). The imputed dataset with INFO > 0.9 shows an optimal prediction of true LoF variants, because it has less false positive 2-copy LoF variants (~ three variants) compared to the others (0.1 and 0.3). However, selecting an optimal imputed allele probability was difficult due to the lack of apparent variations.

Compound heterozygous LoF variants

Compound heterozygous (CH) variants occur when both parents donate a single LoF allele to proband at distinct loci within the same gene [6]. LoFTK can annotate CH LoF variants, which introduce 2 inactive copies of genes. We used trio-families data from the Genome of the Netherlands (GoNL) study (Illumina Immunochip microarray SNP data) [5] to validate the transmitting of real CH LoF variants from parents to probands. We included 760 samples from the GoNL cohort, which they are family-based trios. We excluded variants with a call rate ≤ 0.99, Hardy-Weinburg equilibrium (HWE) ≤ 0.001 and monomorphic variants. After data filtrations, we used the TOPMed imputation server to impute missing genotypes [7]. As a post-imputation quality control step, we excluded variants with imputation quality score < 0.3 (INFO < 0.3) and monomorphic variants.

We utilized LoFTK to predict LoF variants and genes in the imputed genotypes of 250 families. We discovered 250 CH LoF variants generating 2-copy LoF genes in 164 probands. There were 240 (96%) genuine transmissions of CH LoF in parent-offspring with 10 false transmissions.

Supplementary Tables

Imputed allele probabilities	info score					
	0.3		0.6		**0.9**	
	False negative	False positive	False negative	False positive	False negative	False positive
0.01	21.69	19.59	22.18	19.24	23.63	16.42
0.02	21.40	19.78	21.91	19.38	23.42	16.47
0.03	21.23	19.90	21.75	19.48	23.30	16.49
0.04	21.10	19.99	21.64	19.54	23.22	16.51
0.05	21.01	20.06	21.55	19.59	23.16	16.51
0.06	20.90	20.14	21.46	19.64	23.09	16.52
0.07	20.81	20.21	21.38	19.69	23.04	16.54
0.08	20.74	20.27	21.33	19.74	23.00	16.55
0.09	20.68	20.33	21.27	19.79	22.96	16.56
0.1	20.61	20.40	21.22	19.84	**22.92**	**16.58**

Supplementary Table 1: False positive and false negative values from matching the LoF variants between exome and subgrouped imputed genotypes data for each individual.

**Qualitative trait**	**1-copy carriers**	**2-copy carriers**	**CH (%)**	**gene_symbol**	**N_individuals**	**N_eff**	**beta**	**se**	**t**	**pvalue**
LDL_Cholesterol	263	1	1 (100)	PCSK9	42352	71	-0.411	0.050	-8.205	2.37E-16
Alkaline_phosphatase	76970	21755	8764 (40.28)	ABO	159450	115027	-4.606	0.092	-50.012	1.16E-545
Height	41115	3447	27 (0.78)	H6PD	166643	47916	0.176	0.031	5.657	1.54E-08
Height	46260	4601	2290 (49.77)	CPNE1	166643	55353	0.214	0.029	7.300	2.90E-13
BMI	80115	27235	14 (0.05)	ATXN2L	166446	134143	0.115	0.017	6.934	4.10E-12
Total_Triglycerides	30306	1748	5 (0.29)	LPL	42352	8493	-0.102	0.006	-16.141	1.96E-58
**Binary trait**	**1-copy carriers**	**2-copy carriers**	**CH (%)**	**gene_symbol**	**N_individuals**	**N_eff**	**OR**	**CI**	**z**	**pvalue**
hypothyroidism	10801	156	101 (64.74)	IFIH1	125668	8252	-0.281	0.050	-5.604	2.10E-08
hypothyroidism	83546	40669	21 (0.05)	CLECL1	125668	124085	-0.100	0.016	-6.204	5.49E-10
asthma	82228	36710	52 (0.14)	GSDMB	125692	116864	-0.109	0.011	-9.880	5.09E-23

Supplementary Table 2: Association of selected positive control LoF genes with UKBB phenotypes.

Supplementary Figure

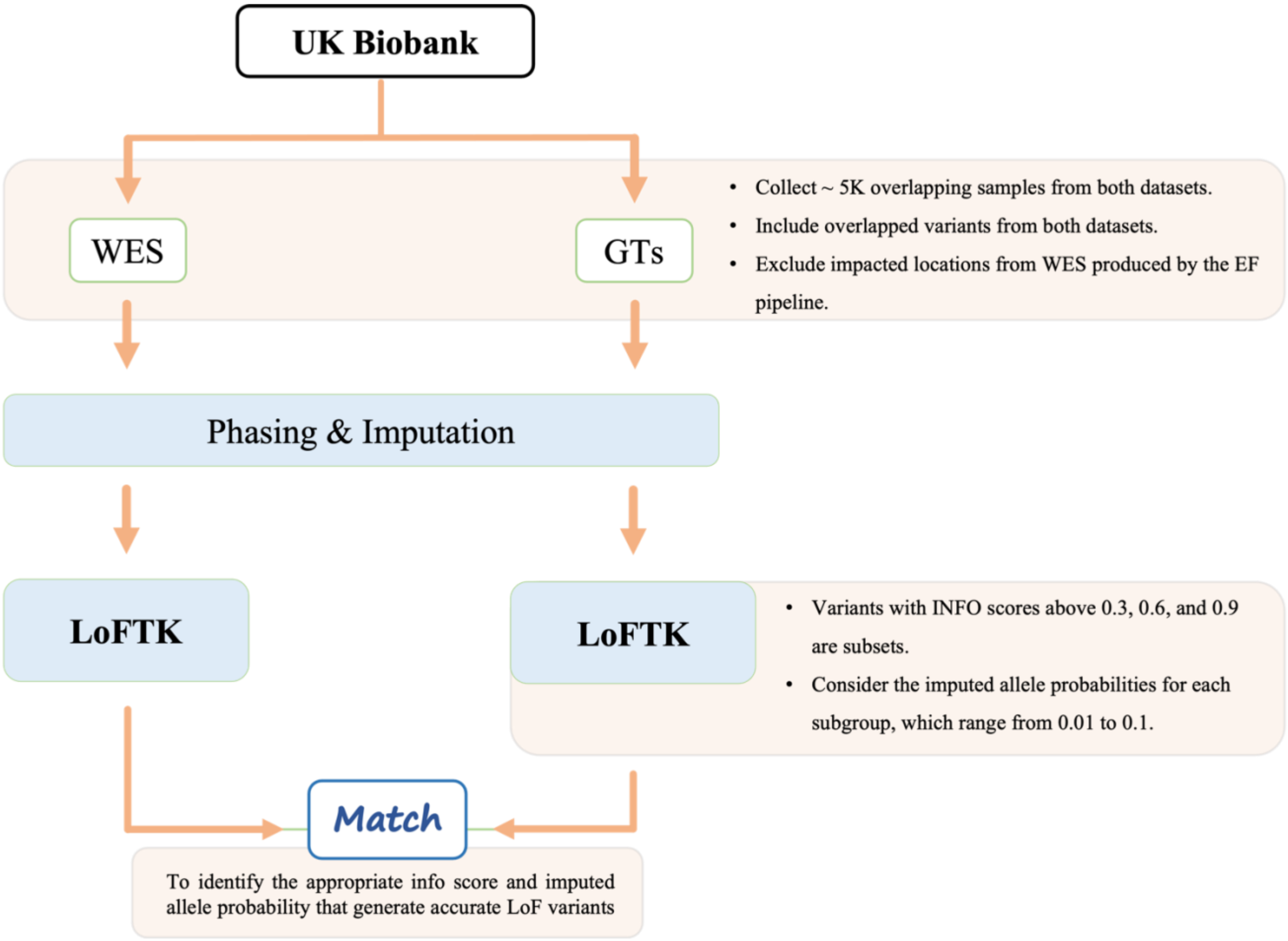

Supplementary Figure 1: The workflow for achieving the optimal imputation quality measure.

References

1. Howie BN, Donnelly P, Marchini J (2009) A flexible and accurate genotype imputation method for the next generation of genome-wide association studies. PLoS Genet 5:e1000529

2. Howie B, Fuchsberger C, Stephens M, Marchini J, Abecasis GR (2012) Fast and accurate genotype imputation in genome-wide association studies through pre-phasing. Nat Genet 44:955–959

3. Delaneau O, Marchini J, Zagury J-F (2011) A linear complexity phasing method for thousands of genomes. Nat Methods 9:179–181

4. 1000 Genomes Project Consortium, Abecasis GR, Auton A, Brooks LD, DePristo MA, Durbin RM, Handsaker RE, Kang HM, Marth GT, McVean GA (2012) An integrated map of genetic variation from 1,092 human genomes. Nature 491:56–65

5. Boomsma DI, Wijmenga C, Slagboom EP, et al (2014) The Genome of the Netherlands: design, and project goals. Eur J Hum Genet 22:221–227

6. Kamphans T, Sabri P, Zhu N, Heinrich V, Mundlos S, Robinson PN, Parkhomchuk D, Krawitz PM (2013) Filtering for compound heterozygous sequence variants in non-consanguineous pedigrees. PLoS One 8:e70151

7. Taliun D, Harris DN, Kessler MD, et al (2021) Sequencing of 53,831 diverse genomes from the NHLBI TOPMed Program. Nature 590:290–299
